# *Notes from the Field:* Multiple Cyclosporiasis Outbreaks — United States, 2018

**DOI:** 10.15585/mmwr.mm6739a6

**Published:** 2018-10-05

**Authors:** Shannon M. Casillas, Carolyne Bennett, Anne Straily

**Affiliations:** 1 Parasitic Diseases Branch, Division of Parasitic Diseases and Malaria, Center for Global Health, CDC.

Cyclosporiasis is an intestinal illness caused by the parasite *Cyclospora cayetanensis* through ingestion of fecally contaminated food or water. Symptoms of cyclosporiasis might include watery diarrhea (most common), loss of appetite, weight loss, cramping, bloating, increased gas, nausea, and fatigue. Typically, increased numbers of cases are reported in the United States during spring and summer; since the mid-1990s, outbreaks have been identified and investigated almost every year. Past outbreaks have been associated with various types of imported fresh produce (e.g., basil, cilantro, and raspberries) (*1*). There are currently no validated molecular typing tools* to facilitate linking cases to each other, to food vehicles, or their sources. Therefore, cyclosporiasis outbreak investigations rely primarily on epidemiologic data.

The 2018 outbreak season is noteworthy for multiple outbreaks associated with different fresh produce items and the large number of reported cases. Two multistate outbreaks resulted in 761 laboratory-confirmed illnesses. The first outbreak, identified in June, was associated with prepackaged vegetable trays (containing broccoli, cauliflower, and carrots) sold at a convenience store chain in the Midwest; 250 laboratory-confirmed cases were reported in persons with exposures in three states (illness onset mid-May–mid-June) (*2*). The supplier voluntarily recalled the vegetable trays (*3*). The second multistate outbreak, identified in July, was associated with salads (containing carrots, romaine, and other leafy greens) sold at a fast food chain in the Midwest; 511 laboratory-confirmed cases during May–July occurred in persons with exposures in 11 states who reported consuming salads (*4*). The fast food chain voluntarily stopped selling salads at approximately 3,000 stores in 14 Midwest states that received the implicated salad mix from a common processing facility (*5*). The traceback investigation conducted by the Food and Drug Administration (FDA) did not identify a single source or potential point of contamination for either outbreak.

In addition to the multistate outbreaks, state public health authorities, CDC, and FDA investigated cyclosporiasis clusters associated with other types of fresh produce, including basil and cilantro. Two basil-associated clusters (eight confirmed cases each) were identified among persons in two different states who became ill during June. Investigation of one cluster, for which the state health department conducted an ingredient-specific case-control study, found consumption of basil to be significantly associated with illness. A formal analytic study was not conducted for the other cluster, but all patients reported consuming basil. Three clusters associated with Mexican-style restaurants in the Midwest have resulted in reports of 53 confirmed cases in persons who became ill during May–August. Analytic studies were conducted for two clusters; consumption of cilantro was found to be significantly associated with illness in both. Although a formal analytic study was not possible for the third cluster, all 32 identified patients reported consuming cilantro at the restaurant. FDA traceback of the basil and cilantro from these clusters is ongoing. Additional clusters associated with Mexican-style restaurants were identified in multiple states; but investigations to determine a single vehicle of infection were unsuccessful because of small case counts, limited exposure information, or because fresh produce items (including cilantro) were served as components of other dishes (e.g., in salsa).

Many cases could not be directly linked to an outbreak, in part because of the lack of validated molecular typing tools for *C. cayetanensis.* As of October 1, 2018, a total of 2,299 laboratory-confirmed cyclosporiasis cases^†^ have been reported by 33 states in persons who became ill during May 1–August 30 and did not have a history of international travel^§^ during the 14 days preceding illness onset. Approximately one third of these cases were associated with either the convenience store chain outbreak or the fast food chain outbreak (Figure). The median patient age was 49 years (range = <1–103 years) and 56% were female (1,288 of 2,285). At least 160 patients were hospitalized; no deaths have been reported.

**FIGURE:**
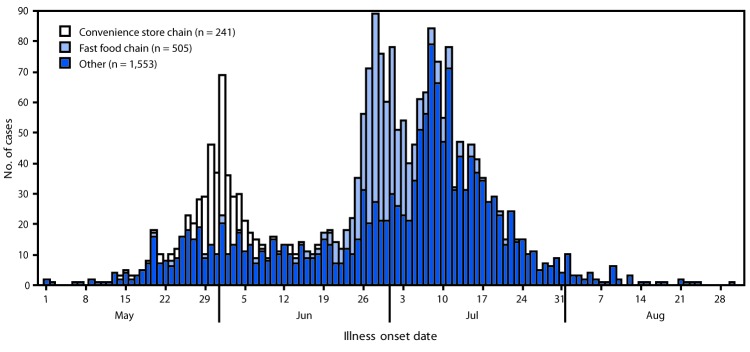
Reported cases of laboratory-confirmed, nontravel-associated cyclosporiasis, by illness onset date and outbreak association — United States, May–August, 2018*^,^^†^ * N = 2,299. Data are current as of October 1, 2018 (1 p.m. EDT). Data are preliminary and subject to change. These cases occurred in persons with no history of travel outside of the United States or Canada in the 14 days before onset of illness. ^†^ Case counts for outbreaks differ from what was posted in the text because of incomplete reporting of travel history (convenience store chain, n = 7; fast food chain, n = 6) or illness onset date (convenience store chain, n = 2).

The 2,299 domestically acquired, laboratory-confirmed cases reported in persons who became ill during May–August 2018 are markedly higher than the numbers of cases reported for the same period in 2016 (174) and 2017 (623). This increase might be due, in part, to changes in diagnostic testing practices, including increased use of gastrointestinal molecular testing panels.^¶^ CDC is working with state public health partners to determine whether and to what extent changes in testing practices might have contributed to increased case detection and reporting.

Consumers should continue to enjoy fresh produce as part of a well-balanced diet. To reduce risk from most causes of foodborne illness and other contaminants, CDC recommends washing fresh fruits and vegetables with clean running water; however, washing, including use of routine chemical disinfection or sanitizing methods, is unlikely to kill *C. cayetanensis*. Persons with diarrheal illness that lasts >3 days or who have any other concerning symptoms should see a health care provider if they think they might have become ill from eating contaminated food.

## Footnotes

* CDC scientists are currently working to develop and validate genetic typing markers for *Cyclospora*; such markers could facilitate linking cases of cyclosporiasis to each other and to food vehicles and their sources.

† For 2018, the numbers of cases reported by states might change as investigations are finalized.

§ International travel was defined as travel outside of the United States or Canada.

¶ Historically, the standard laboratory method used for diagnosing *Cyclospora* infection was an Ova and Parasite (O&P) examination; routine O&P examination typically does not detect *Cyclospora*, so health care providers must specifically request testing for the parasite. In 2014, the first FDA-cleared gastrointestinal polymerase chain reaction panel with a target for *Cyclospora* became commercially available; this testing method is more sensitive than traditional O&P examination and has the potential to improve case detection.
